# Walking and taking vitamin C alleviates oxidative stress and inflammation in overweight students, even in the short-term

**DOI:** 10.3389/fpubh.2022.1024864

**Published:** 2022-10-05

**Authors:** Qian Zhang, Miao Guo, Tianyi Chen, Huizhi Cheng, Qianwen Yang, Zhuohui Zhao, Rong She, Xiaoyan Yang, Wen Xiao, Xu Yang, Lijuan Li

**Affiliations:** ^1^Institute of Natural Antioxidants and Antioxidant Inflammation, Dali University, Dali, China; ^2^School of Public Health, Dali University, Dali, China; ^3^Joint International Research Laboratory of Green Buildings and Built Environments (Ministry of Education), Chongqing University, Chongqing, China; ^4^Department of Environmental Health, School of Public Health, Fudan University, Shanghai, China; ^5^Institute of Eastern-Himalaya Biodiversity Research, Dali University, Dali, China; ^6^Xianning Medical College, Hubei University of Science and Technology, Hubei, China

**Keywords:** overweight/obesity, oxidative stress, inflammatory cytokine, alleviation, walking, vitamin C, intervention, panel study

## Abstract

**Objective:**

Obese or overweight is a risk factor for some chronic diseases, and oxidative stress and inflammation may be one of the molecular mechanisms leading to the persistence of these chronic diseases. Discovering interventions to alleviate oxidative stress and inflammation in the overweight/obese population, is very important for public health and health education.

**Methods:**

A two-week panel intervention study (Run 0-Run 1-Run 2) was conducted. The subjects were 77 overweight/obese undergraduates attending Dali University, with a BMI>24 kg/m^2^. The physical indices measured at the end of each run included BMI, waist circumference, serum ROS, TNF-α, IL-1β and urinary 8-OHdG. Students were allocated to one of four intervention groups: No intervention (control); walking; taking vitamin C; and walking + taking vitamin C.

**Results:**

The results demonstrated (1) Walking significantly alleviated ROS levels, and this was consistent in Run 1 and Run 2; (2) During Run1, all three intervention modes reduced levels of 8-OHdG, but there was a statistically insignificant increase during Run 2; (3) No alleviating effects of the three intervention modes on TNF-α levels during Run 1 and Run 2 were observed; (4) The alleviating effects of the three intervention modes on IL-1β levels during Run 1 and Run 2 were clear.

**Conclusion:**

Walking and taking vitamin C can reduce levels of ROS, 8-OHdG and IL-1β, but not TNF-α, in overweight/obese participants. These interventions may become potential preventive measures for the overweight against obese-induced oxidative stress and inflammation.

## Introduction

In 1984, the world-famous economist and professor at Harvard University, John Kenneth Galbraith, pointed out that increasingly Americans were dying not from a lack of food, but rather from excess food (energy intake) (1). This view is now widely recognized by American biomedical scholars (2). In addition, the World Health Organization has identified obesity to be one of the top 10 chronic diseases, being the second most common cause of death, only to smoking in today's society. According to the findings of current scientific research, excess energy intake can not only cause overweight/obesity, but can also cause oxidative stress/oxidative inflammation (3). The combined effect of obesity and oxidative inflammation may be an important factor in the persistence of many chronic diseases.

After more than 40 years of reform and open, and rapid economic development, China is now facing the same challenge where excess energy intake has become an important risk factor for national health. According to results from the latest survey conducted by China's National Health Commission on December 23, 2019, the percentage of overweight adults (BMI ≥ 24) in China is 50.7%, or nearly 500 million adults; the percentage of obese adults (BMI ≥ 28) is more than 16.4%, ~150 million adults. In fact, China has become a veritable “fat country.”

Relevant studies have shown that: compared with normal-weight people, the serum vitamin C concentration in overweight and obese people is significantly lower, and BMI is negatively correlated with vitamin C concentration (4, 5). Some scholars believe that the emergence of this phenomenon may be related to the increased production of ROS in overweight and obese people (6), depleting the storage of antioxidants including vitamin C. Vitamin C plays an important role in scavenging free radicals and inhibiting lipid peroxidation. The mechanism underlying the antioxidant, anti-inflammatory properties of vitamin C has been attributed to its ability to modulate nuclear factor kappa-B (NF-κB) transcriptional activation gene in DNA binding site activity (7). The activation of NFκB proinflammatory gene in DNA can be promoted by oxidative stress. At the same time, it leads to the expression of vascular endothelial cytokine-induced cell adhesion molecule (CAM) molecule, and the production of hepatic TNF-α and IL-6-induced C-reactive protein (CRP) (8).

Since the beginning of the 21st century, due to the rapid development of biomedicine, especially the widespread use of antibiotics, infectious diseases have been significantly reduced. Therefore, the probability of generating ROS through the “pathogenic microbes phagocytic pathway” has been significantly reduced, and the number of generated ROS has been greatly decreased. Moreover, the environmental pollutants entering the body are gradually decreasing, with the gradual improvement of China's environmental quality. It can be expected that ROS generated by the “endoplasmic reticulum cytochrome P450 reactions” in the Chinese is also gradually decreasing. Therefore, a hypothesis (3) is proposed: ROS in the body comes from excess energy intake, and in a relatively good and pollution-free environment, if the body is not attacked by pathogenic microorganisms. In other words, oxidative damage/oxidative inflammation is a downstream parallel pathological event of excess energy intake. Exercise can increase the body's energy expenditure. Antioxidants can act as blockers to interfere with the overproduction of ROS in the body.

With this background, our panel intervention study aims to explore whether exercise and the use of appropriate antioxidants can alleviate the level of oxidative stress/inflammation in the overweight/obese population in a short period, so as to provide relevant scientific data for effective health intervention and health education in the overweight/obese population in the future.

## Materials and methods

### Participants

This project used the principle of cluster sampling to recruit research participants at Dali University, Yunnan Province. The selection conditions of recruitment were: (1) freshmen and sophomores of the university; (2) they were overweight students with a BMI ≥ 24, which included obese students (BMI ≥ 28); (3) non-smokers; (4) agreed to complete an informed consent form. Exclusion criteria for participants included: (1) minors under the age of 18 (because they cannot sign the informed consent form by themselves); (2) overweight or obese students suffering from other diseases, especially those with infectious diseases (to avoid oxidative stress/oxidative inflammation caused by pathogenic microorganism infection confusing the results).

The number of study participants was determined by combining the overweight and obesity rates among freshmen and sophomores of the university. The number of investigators was finally determined to be no < 15 people in each group who finally completed the intervention. In the end, a total of 103 students were recruited. The research participants were randomly sampled and entered into the no intervention group, healthy exercise intervention group, antioxidant intervention group, and healthy exercise and antioxidant combined intervention group in sequence. Of the original pool of 103 students, 21 were eliminated according to the recruitment conditions, so 82 participants actually entered the study, 5 withdrew halfway, leaving 77 to complete the study.

### Methods

#### Panel study design

**Figure 1:**
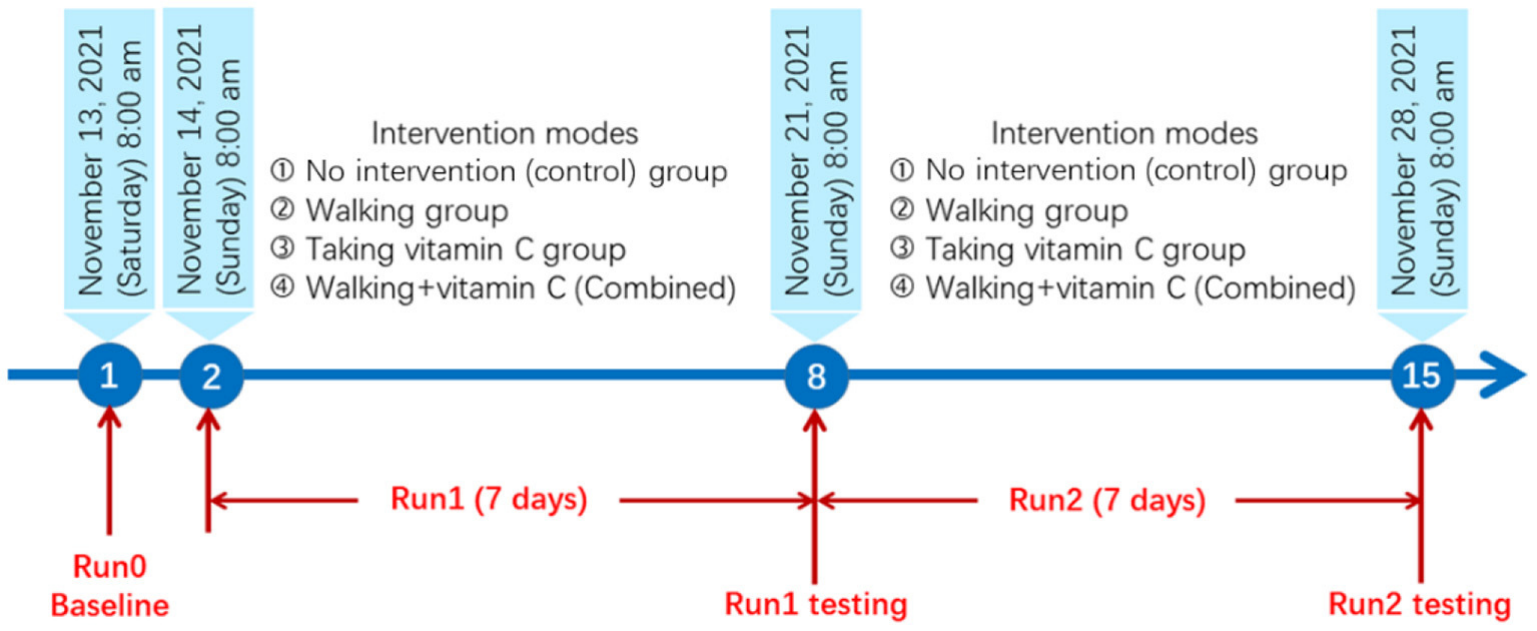
The research protocol for this panel study.

The panel study lasted 15 days from November 13 to November 28, 2021. The detailed research protocol is shown in Figure 1.

#### Intervention groups

There were four treatment groups: (1) no intervention group (control group): these participants had no intervention measures, and maintained their lifestyle, diet and exercise habits as before the start of this study; (2) healthy exercise intervention group (walking group): in addition to maintaining the same lifestyle and eating habits as before the study, these participants walked 6,000 steps (9) every afternoon in the playground of Dali University (steps were recorded by a 5S-Model Lexin Intelligent Exercise Bracelet from Guangdong Lexin Medical Electronics Co., Ltd.); (3) antioxidant intervention group (vitamin C group): participants in this group took 300 mg of vitamin C (9) every day (100 mg after breakfast, lunch and dinner) in addition to maintaining the same lifestyle, diet and exercise habits as before the study; (4) healthy exercise and antioxidant combined intervention group (walking + vitamin C group): in addition to maintaining the same lifestyle and eating habits as before this study, the participants in this group also walked 6,000 steps every afternoon alongside the walking group using the same step counting method, and took 300 mg of vitamin C every day (100 mg of vitamin C after each meal).

#### Indicators and measurement

We examined the following three groups of indicators: (1) overweight or obesity, namely body mass index (BMI) and waist circumference; (2) biomarkers of oxidative stress, including serum ROS and urine 8-OHdG levels; (3) biomarkers of inflammation, including serum TNF-α and serum IL-1β levels. The levels of oxidative stress and inflammation were measured using human Enzyme-Linked Immunosorbent Assay (ELISA) kits from Jiangsu Jingmei Biotechnology Co., Ltd of China. The biomarker tests started at 8:00 am on Day 1, Day 8, and Day 15.

#### Grouping and quality control

The participants were randomly assigned to one of the above four groups by computer lottery. The on-site staff engaged in this study were specially trained in advance at the School of Public Health of Dali University, to unify the diagnostic criteria and survey introduction, to ensure the quality of epidemiological investigation data. The obtained questionnaires were coded by a specially assigned person, and computer input and backup was done after verification. The laboratory testing method was carried out according to detailed operation instructions, quality control was implemented in the testing process, and the research information and data were backed up by dual computers.

#### Informed consent

The project strictly adhered to the Helsinki Convention of ethical principles for medical research involving human participants issued by the World Medical Association. After recruitment of research participants, they were given a unified and informed explanation of the study, introducing the research background and scientific significance, research process and methods, possible benefits to the research participants, possible risks (zero risk), privacy issues, costs and compensation, the right to withdraw freely, etc. The research participants then were asked to sign the informed consent form.

#### Informed consent statement

Informed consent was obtained from all participants involved in the study.

#### Ethical standards disclosure

This study was conducted according to the guidelines laid down in the Declaration of Helsinki and all procedures involving research study participants were approved by the Medical Ethics Committee of Dali University (protocol code: MECDU-202103-7 and date of approval: 2021/3/15). Written and verbal informed consent was obtained from all participants and legal guardians.

### Statistical analysis

Statistical analysis included three different analyses: (1) basic statistical analysis (Office-Excel 2016) of baseline indicators, including the overall and gender mean, standard deviation and sample size; (2) an ANOVA analysis (GraphPad Prism 7.0 software) of oxidative stress and inflammatory biomarkers, that included a repeated measures one-way ANOVA, (RM-ANOVA) for repeated measurements taken during the intervention process, and an ordinary one-way ANOVA, (O-ANOVA) for data from the different intervention groups. The multiple comparison test used for the analysis of variance was Dunnett's test; (3) mixed effects model analysis (lme4 package of R software v4.0.4), the data from the no intervention group were excluded from this analysis; the logarithm of the biomarker results of the walking group, the vitamin C group and the combined intervention group was taken as a fixed factor; age, gender, BMI, and waist circumference were used as covariants of the fixed factor; student ID was used as a random factor. These statistical analyses were all carried out by a two-sided test, and a level of significance α = 0.05, was set for all statistical tests.

Due to the influence of changes in laboratory environmental conditions, there were systematic differences between the ELISA measurement results for oxidative stress and inflammation biomarkers for the three repeated measurements (in research course: Run0, Run1, and Run2), so the average values from the no intervention group were used as the denominator for normalization (results expressed in %), to observe the endpoint effects of the oxidative stress and inflammation biomarkers.

## Results

### Baseline data

The research participants of this study were all overweight undergraduate students, with a BMI ≥ 24 kg/m^2^; a total of 77 students took part in, in whom 45 were overweight but not obese (24 < BMI < 28), and 32 were obese (BMI>28). The students were randomly assigned to one of the four intervention groups: 17 students in the no intervention group, 16 in the walking group, 24 in the vitamin C group, and 20 in the combined intervention group. The participants of this study are freshmen and sophomores of the School of Basic Medicine of Dali University, whose study arrangements and living routines are similar. Due to the school being closed during the intervention period, the research participants ate three meals a day in the school cafeteria (the participants were asked not to order takeout during the intervention period). The groups therefore had a degree of consistency in eating and sleeping, allowing for a balance between groups.

### Oxidative stress and inflammatory biomarkers

Our panel intervention design called for analyses of variance for two factors: among the different intervention courses (Run 0-Run 1-Run 2), we used the repeated measures one-way ANOVA (RM-ANOVA); among the different intervention groups, we used the ordinary one-way ANOVA (O-ANOVA). The results are shown in **Table 2**.

**Figure 2:**
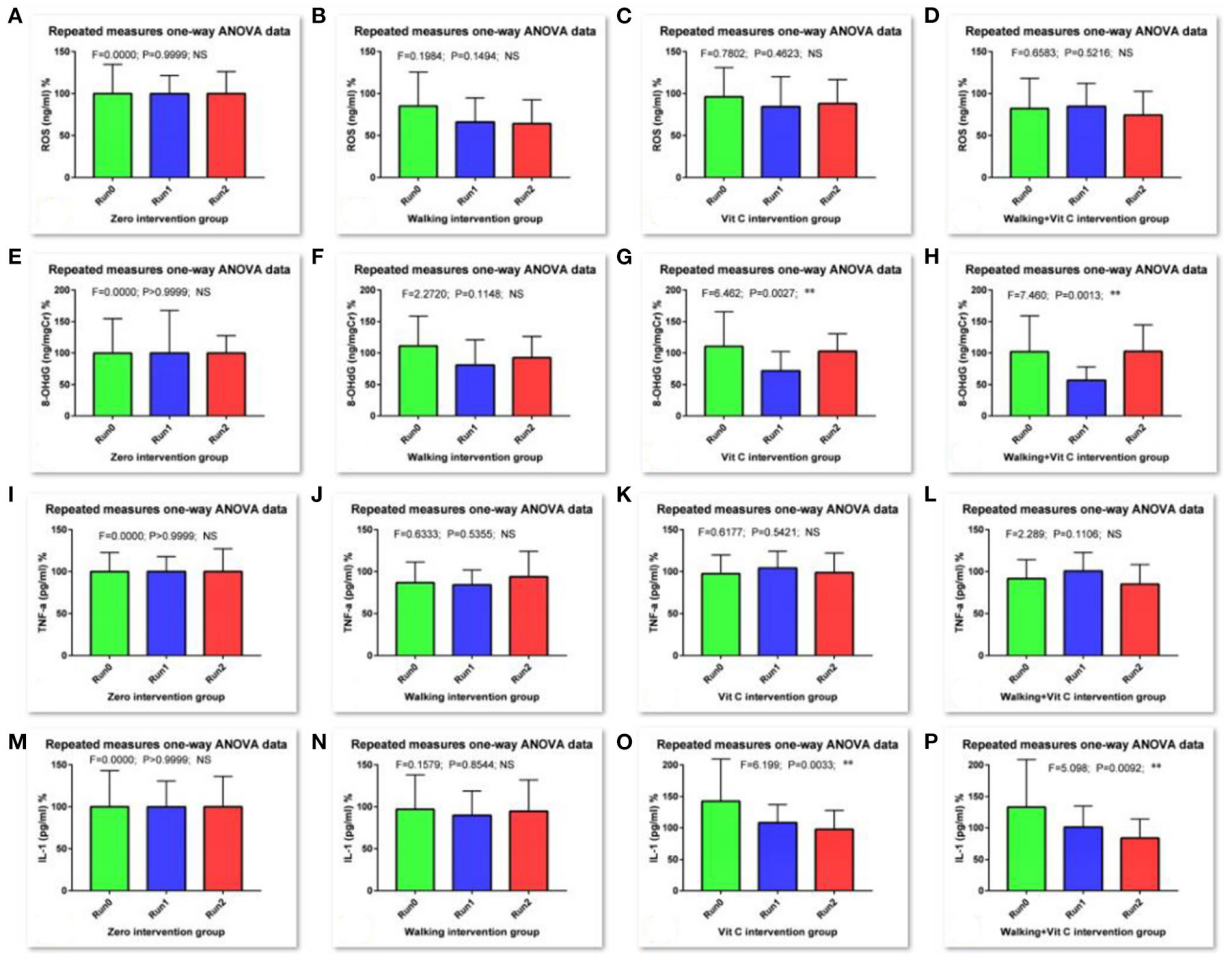
Results of RM-ANOVA. [**(A–D)** are for ROS in Run0, Run1 and Run2 groups, **(E–H)** are for 8-OHdG in Run0, Run1 and Run2 groups, **(I–L)** are for TNF-α in Run0, Run1 and Run2 groups, and **(M–P)** are for IL-1β in Run0, Run1 and Run2 groups. Comparison for research courses with RM-ANOVA, *: the difference is significant (*p* < 0.05); **: the difference is very significant (*p* < 0.01)].

As we can see from Table 1 and Figure 2: the RM-ANOVA results show that the serum ROS (an oxidative stress biomarker) levels when compared with the levels of the baseline survey (Run 0), all decreased during Run 1 and Run 2, except for the no intervention group, but this decrease was not significant. The results of the O-ANOVA analysis showed that, compared with the no intervention group, the decrease in serum ROS levels in the walking group was very significant in the 1st and 2nd weeks (*p* < 0.01); and compared with the no intervention group, the decrease in serum ROS levels of the combined intervention group (walking + vitamin C) was significant in the 2nd week (*p* < 0.05). These results show that walking can reduce the level of serum ROS over a short period.

**Table 1:** Statistical data of baseline measurement.

**Indicators**	**Measurements (mean** ±**Sd)**		
	**Male (*N* = 51)**	**Female (*N* = 26)**	**Total (*N* = 77)**
Age (year)	19.08 ± 0.98	19.08 ± 0.93	19.08 ± 0.96
BMI (kg/m^2^)	27.29 ± 2.60	27.96 ± 2.57	27.52 ± 2.59
Waist circumference (cm)	86.68 ± 5.45^**^	82.81 ± 5.84	85.37 ± 5.84

The t-test,

** : indicated a significant difference between boys and girls (p < 0.01).

We measured the levels of urinary 8-OHdG which is an oxidative stress biomarker. These results analyzed with RM-ANOVA showed that, compared with baseline data levels (Run 0), levels of urinary 8-OHdG in the walking group decreased during Run 1, but not significantly, while levels in the vitamin C group and the combined intervention group decreased very significantly (*p* < 0.01); During Run 2, the urinary 8-OHdG levels in the vitamin C group and the combined intervention group increased slightly, but this difference was not significant. The results of O-ANOVA analysis showed that the urinary 8-OHdG levels in the combined intervention group decreased significantly after the 1st week of intervention compared with the no intervention group for the same period (*p* < 0.01). The analysis also showed that vitamin C had a certain attenuating effect on levels of urinary 8-OHdG in a short period.

Levels of serum TNF-α, a biomarker of inflammation, were determined in this study. RM-ANOVA analysis showed that, compared with baseline data (Run 0), with the exception of the no intervention group, levels of serum TNF-α in the other three intervention groups were significantly affected during Run 1 and Run 2. The serum TNF-α levels either decreased slightly or increased slightly, with just one difference reaching a significant level. The results of O-ANOVA analysis showed that, compared with the no intervention group, the various interventions of the other three groups had no significant effect on serum TNF-α levels after the interventions during the 1st and 2nd weeks. This means that walking and vitamin C intervention reduced serum TNF-α, but this effect was not obvious in this short period.

We also measured levels of serum IL-1β (another inflammation biomarker). The RM-ANOVA showed that levels of IL-1β in the vitamin C group decreased during Run1 when compared with baseline data levels (Run 0) (*p* < 0.05); during Run 2, levels of serum IL-1 β decreased in both the vitamin C intervention group and the combined intervention group, and this alleviating effect of vitamin C on serum IL-1β levels was very significant (*p* < 0.01), indicating that vitamin C intervention has a certain effect on reducing serum IL-1β levels over a short period. The results of O-ANOVA analysis showed that, compared with the no intervention group for the same period, serum IL-1β levels decreased after baseline measurement and week 1 intervention. The levels increased and decreased slightly after the 2nd week, but these differences were not significant.

### Mixed effects model analysis for oxidative stress and inflammation biomarkers

**Figure 3:**
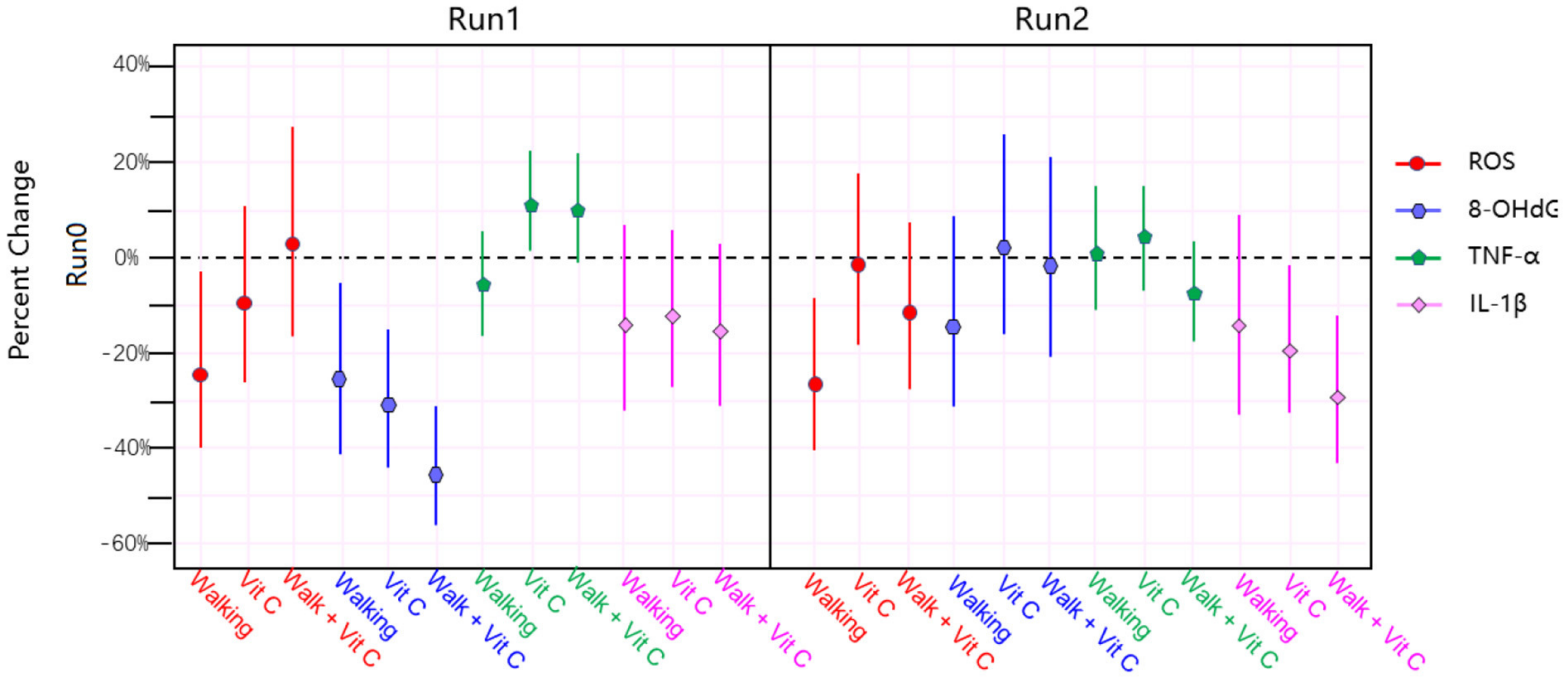
The results of linear mixed effects model analysis.

The results of the analysis are shown in Figure 3 and Table 2.

**Table 2:** Results of ANOVA-Dunnett's test of oxidative stress and inflammatory biomarkers.

**Biomarkers**	**Research courses**	**Intervention modes (groups)**			
		**No intervention** **(*N* = 17)**	**Walking** **(*N* = 16)**	**Vit. C** **(*N* = 24)**	**Walking + Vit. C** **(*N* = 20)**
Serum ROS (ng/ml)%	Run 0	100.00 ± 34.86	85.31 ± 40.30	96.28 ± 34.64	82.33 ± 35.95
	Run 1	100.00 ± 21.53	66.31 ± 28.63^**##**^	84.60 ± 35.65	85.01 ± 27.43
	Run 2	100.00 ± 26.00	64.28 ± 28.50^**##**^	88.29 ± 28.68	74.28 ± 28.22^**#**^
Urine 8-OHdG (ng/mgCr)%	Run 0	100.00 ± 54.78	111.61 ± 47.08	110.75 ± 54.76	102.01 ± 57.31
	Run 1	100.00 ± 67.62	81.32 ± 39.91	71.77 ± 30.60^**^	56.93 ± 21.08^**^^##^
	Run 2	100.00 ± 27.82	92.66 ± 33.71	102.72 ± 27.99	102.55 ± 42.26
Serum TNF-α (pg/ml)%	Run 0	100.00 ± 22.84	87.08 ± 24.59	97.60 ± 22.29	91.79 ± 22.82
	Run 1	100.00 ± 18.00	84.39 ± 17.36	104.22 ± 20.12	100.62 ± 22.16
	Run 2	100.00 ± 27.22	93.90 ± 30.24	98.79 ± 23.39	85.30 ± 23.16
Serum IL-1β (pg/ml)%	Run 0	100.00 ± 43.17	97.01 ± 41.19	142.43 ± 67.97	132.91 ± 72.12
	Run 1	100.00 ± 30.60	90.01 ± 28.90	108.24 ± 28.67*	101.09 ± 33.65
	Run 2	100.00 ± 36.34	94.86 ± 37.10	97.64 ± 30.32^**^	84.09 ± 29.95^**^

Comparison of research courses: *: compared with Run 0, the difference is significant (p < 0.05);

** : compared with Run 0; the difference is very significant (p < 0.01); Comparison of intervention modes:

# : compared with no intervention group, the difference is significant (p < 0.05);

## : compared with no intervention group, the difference is very significant (p < 0.01).

Figure 2 and Table 3 shows that the three intervention modes (walking, vitamin C and combined intervention) had obvious alleviation effects for Serum ROS, Urine 8-OHdG and Serum IL-1β, but not for serum TNF-α, whose alleviation effect is not obvious. The linear mixed effect model analysis of oxidative stress and inflammation indices agree well-with the results from the analysis of variance.

**Table 3:** Percentage change, 95% confidence interval, and *p*-value range for biomarkers during Run 1 and Run 2 compared to results from Run 0.

**Biomarkers**	**Intervention modes**	**Percent change (%)**	**95% confidence interval (CI %)**		**P value**
			**Lower limit**	**Upper limit**	
**Run 1 vs. Run 0**					
Serum ROS	Walking	−23.69	−39.97	−3.00	0.027
	Vit. C	−9.31	−25.87	10.94	0.342
	Combined	2.73	−17.34	27.67	0.808
Urine 8–OHdG	Walking	−25.53	−41.90	−4.57	0.020
	Vit. C	−30.95	−44.18	−14.59	0.001
	Combined	−45.04	−56.20	−31.03	< 0.001
Serum TNF–α	Walking	−5.52	−15.70	5.90	0.330
	Vit. C	11.28	1.16	22.42	0.028
	Combined	9.97	−0.80	21.92	0.071
Serum IL-1β	Walking	−14.12	−31.50	7.67	0.187
	Vit. C	−12.14	−27.28	6.16	0.180
	Combined	−15.45	−31.08	3.74	0.108
**Run 2 vs. Run 0**					
Serum ROS	Walking	−25.28	−40.07	−6.85	0.010
	Vit. C	−1.89	−18.44	18.00	0.839
	Combined	−11.88	−27.82	7.57	0.214
Urine 8–OHdG	Walking	−13.86	−32.04	9.18	0.217
	Vit. C	3.19	−15.76	26.41	0.762
	Combined	−2.15	−21.22	21.54	0.844
Serum TNF–α	Walking	1.78	−10.54	15.80	0.788
	Taking Vit. C	4.38	−6.30	16.28	0.426
	Combined	−7.83	−17.98	3.57	0.171
Serum IL-1β	Walking	−13.98	−32.23	9.19	0.216
	Vit. C	−19.32	−33.98	−1.41	0.036
	Combined	−29.17	−42.94	−12.07	0.002

In summary: (1) Walking significantly alleviated ROS levels, and this was consistent in Run 1 and Run 2; (2) During Run 1, all three intervention modes reduced levels of 8-OHdG, but there was a statistically insignificant increase during Run 2; (3) No alleviating effects of the three intervention modes on TNF-α levels during Run 1 and Run 2 were observed; (4) The alleviating effects of the three intervention modes on IL-1β levels during Run 1 and Run 2 were clear.

## Discussion

Participants entered this study through strict inclusion and exclusion criteria. All were healthy overweight and obese persons without communicable diseases or chronic diseases. Since other factors that might contribute to obesity or oxidative inflammation were ruled out, excessive energy intake could be the only cause of obesity and oxidative inflammation in this population.

Numerous recent studies of overweight/obese patients have found that levels of inflammatory factors are elevated (10–12), and the levels of inflammatory factors are positively correlated with the severity of obesity (13–15). Obesity has been recognized as a “systemic low-grade inflammatory disease” (16–18). Some other recent studies have found that the level of oxidative stress in overweight/obese patients is also increased (10, 19, 20), and the level of oxidative stress is positively correlated with BMI (21–24). The inflammation caused by oxidative stress/oxidative damage through the continuous activation of the intracellular NFκB pathway is called “oxidative inflammation” (3, 25). Excess energy intake (or “high dietary energy”) is one of the common risk factors for obesity and oxidative inflammation (26–28), so some scholars believe that there is a pathological mirror relationship between obesity and oxidative inflammation, that “they are identical twins” (3).

Obesity-related metabolic syndrome (29–32) and five major chronic diseases [cardiovascular diseases (33–35), neoplasms (36–38), chronic respiratory diseases (39, 40), neurological disorders (41–44), diabetes and kidney diseases (45–48)] may be the biggest public health problems facing China and the world. In 2019, the proportion of deaths caused by these diseases was as high as 66.92% globally, while in China the proportion was 86.21%, meaning incredibly, that < 14% of deaths in China were not caused by these five major diseases (49). Oxidative inflammation in obesity may be one of the main molecular mechanisms leading to the persistence of these major chronic diseases. Worldwide, research into the relationship between oxidative inflammation and these major chronic diseases has begun to receive extensive attention (3). In the future, research into intervention measures to reduce oxidative inflammation will become an important part of improving national health, with important roles for clinical and preventive medicine, and social welfare.

**Figure 4:**
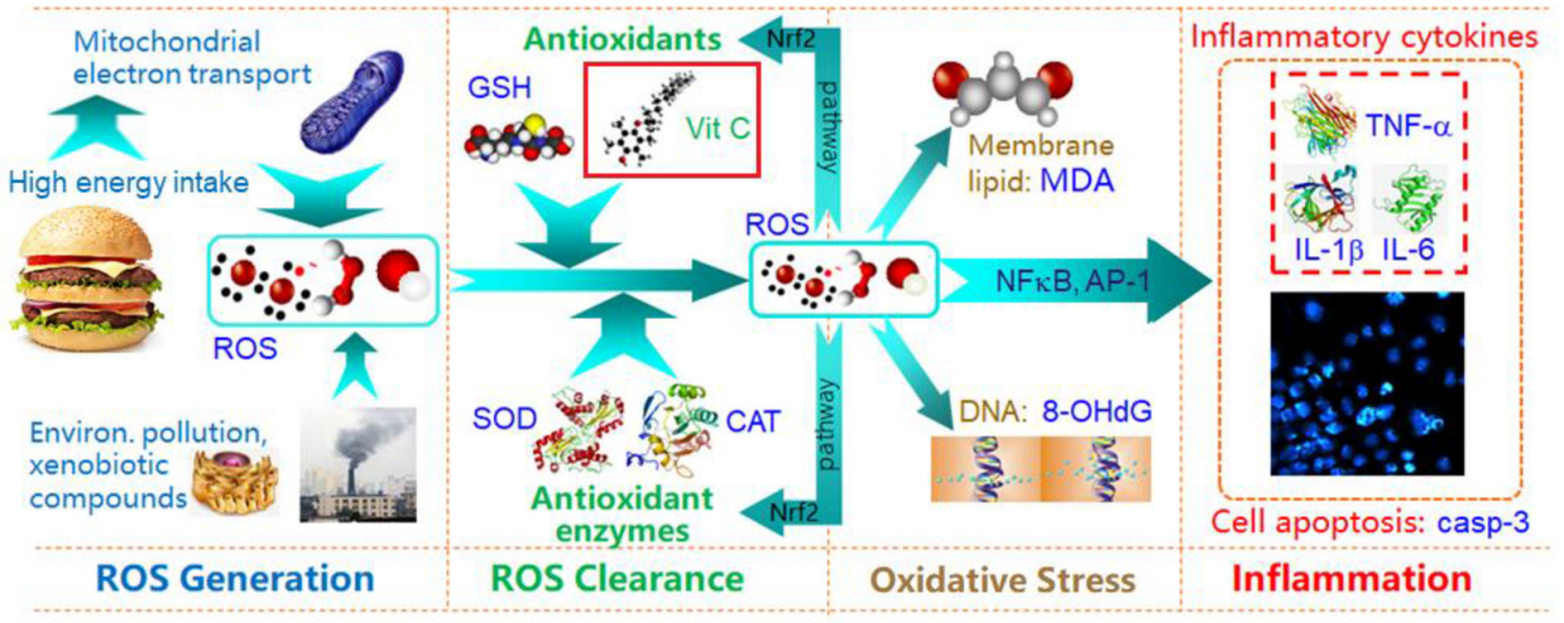
Schematic diagram of energy intake, oxidative stress and inflammation, and Vit C protective effects.

As a pioneering study to explore intervention measures to counter oxidative inflammation, we explore whether healthy exercise (walking), and taking an antioxidant (vitamin C), can reduce the levels of oxidative stress and inflammatory factors in overweight/obese students in the short term. The main physiological mechanism of walking is to maintain energy balance and avoid the accumulation of excess energy in the body, so as to prevent increased electron leakage from cell mitochondria due to an overloaded energy metabolism, and to reduce levels of ROS (50). We used vitamin C in this study because it is a readily available antioxidant nutrient. Figure 4 illustrates how vitamin C (as antioxidant) alleviates oxidative stress and inflammation, and why oxidative stress can induce inflammation. For this panel intervention study, we selected serum ROS and urine 8-OHdG as classical biomarkers of oxidative stress, and serum TNF-α and IL-1β as classical biomarkers of oxidative inflammation. Our study was successfully completed with a Run 0-Run 1-Run 2-2-week intervention, and three repeated sets of tests after each run.

In this study, our main findings were: (1) “Effectiveness” of the intervention measures: Figure 3 shows that the three intervention measures we adopted have obvious alleviating effects on the levels of ROS, 8-OHdG and IL-1β in the participants, and in some cases these alleviating effects were statistically very significant; (2) “Substitutability” of the intervention measures: that is, the alleviating effects of the three interventions are similar, and they are not indispensable to each other. Figure 3 indicates that these interventions have a more obvious effect on IL-1β alleviation; (3) “Non-symbiotic” of the alleviating effects: the main finding is that the combined intervention (walking + vitamin C) did not evince the expected additive effect. The reason for this is worthy of further in-depth study.

This study has some limitations. First, the course of our panel intervention study may not be long enough. In this study, we observed that the level of serum TNF-α has not been alleviated during Run 1 and Run 2. The most likely reason is that 2 weeks is not enough time for the delayed effect of TNF-α to appear. In a future study, we will extend the research course to Run 3 or Run 4. Second, the age range of the participants is very narrow, with the average age and standard deviation being just 19.08 ± 0.96 years. In future work we will recruit overweight people from young, middle-aged and elderly groups, to better understand how age distribution affects the intervention results.

## Conclusions

This study shows that short-term walking exercise and taking vitamin C can effectively alleviate ROS, 8-OHdG and IL-1β levels in overweight/obese college students. However, no effect of intervention measures on serum TNF-α levels was observed in this study. These interventions may become potential preventive measures for the overweight against obese-induced oxidative stress and inflammation.

## Data availability statement

The raw data supporting the conclusions of this article will be made available by the authors, without undue reservation.

## Ethics statement

The studies involving human participants were reviewed and approved by Medical Ethics Committee of Dali University (protocol code: MECDU-202103-7 and date of approval: 2021/3/15). The patients/participants provided their written informed consent to participate in this study.

## Author contributions

Conceptualization, methodology, and formal analysis: XY, LL, WX, XyY, and RS. Software: XY, ZZ, MG, and TC. Validation: XY, QZ, and ZZ. Investigation: QZ, HC, and QY. Resources: LL and WX. Data curation: QZ, XY, MG, and TC. Writing-original draft preparation: XY, LL, MG, and QZ. Writing-review and editing: WX, XyY, ZZ, and RS. Visualization: XY and MG. Supervision: XY and LL. Project administration: LL and QZ. Funding acquisition: LL, XY, and RS. All authors have read and agreed to the published version of the manuscript.

## Funding

This research was funded by: (1) Science and technology planning projects of Yunnan Provincial Department of Science and Technology (202101BA070001-115), (2) Special Basic Cooperative Research Programs of Yunnan Provincial Undergraduate Universities' Association (202001BA070001-065), and (3) National Natural Science Foundation of China (21577045).

## Conflict of interest

The authors declare that the research was conducted in the absence of any commercial or financial relationships that could be construed as a potential conflict of interest.

## Publisher's note

All claims expressed in this article are solely those of the authors and do not necessarily represent those of their affiliated organizations, or those of the publisher, the editors and the reviewers. Any product that may be evaluated in this article, or claim that may be made by its manufacturer, is not guaranteed or endorsed by the publisher.
