# Supplementary figures and images for: Walking and taking vitamin C alleviates oxidative stress and inflammation in overweight students, even in the short-term
Source: Front Public Health. 2022 Oct 5;10:1024864. doi: 10.3389/fpubh.2022.1024864 (PMC9581260; doi:10.3389/fpubh.2022.1024864)

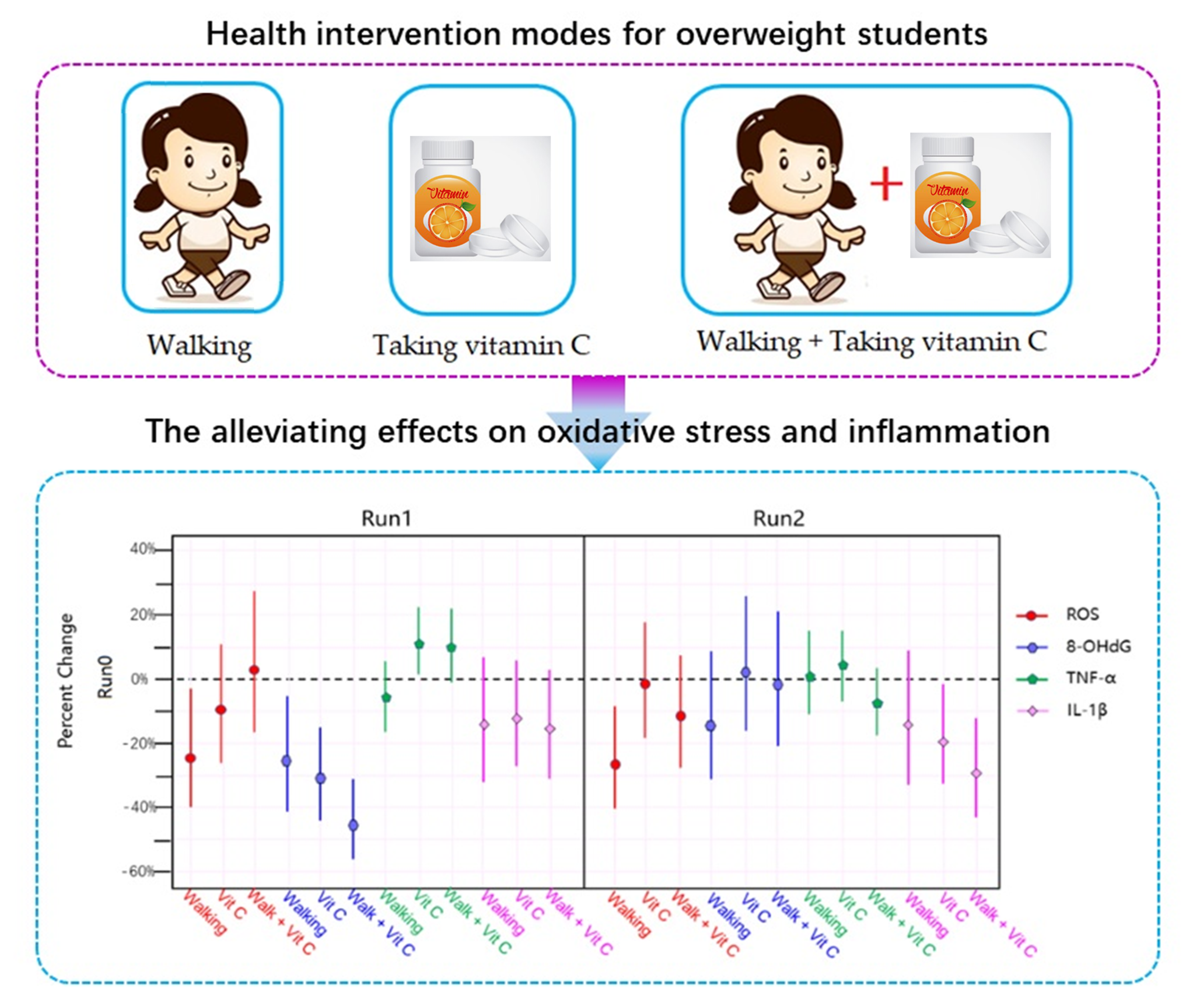

Supplement: Supplementary file 1 [file Image_1.TIF]
